# Exploring the effects of seasons, diurnal cycle, and heights on airborne pollen load in a Southeast Asian atmospheric condition

**DOI:** 10.3389/fpubh.2022.1067034

**Published:** 2022-12-14

**Authors:** Yotin Juprasong, Sirin Sirirakphaisarn, Umaporn Siriwattanakul, Wisuwat Songnuan

**Affiliations:** ^1^Graduate Program in Toxicology, Faculty of Science, Mahidol University, Bangkok, Thailand; ^2^Center of Excellence on Environmental Health and Toxicology (EHT), Office of the Permanent Secretary (OPS), Ministry of Higher Education, Science, Research and Innovation (MHESI), Bangkok, Thailand; ^3^Systems Biology of Diseases Research Unit, Faculty of Science, Mahidol University, Bangkok, Thailand; ^4^Department of Plant Science, Faculty of Science, Mahidol University, Bangkok, Thailand

**Keywords:** pollen allergy, airborne pollen, pollen dynamics, meteorological factors, tropical/subtropical plants

## Abstract

**Introduction:**

Aeropollen can induce detrimental effects, particularly in respiratory airways. Monitoring local aeropollen is essential for the management of pollen allergic patients in each area. However, without resources for constant monitoring, pollen counts are subjected to biases imposed by the choices of sampling season, time of collection, and location. Therefore, the effects of these factors must be better understood. This study investigated the dynamics of aeropollen types through seasonal variation, diurnal cycle and different heights from the ground in Bangkok, Thailand.

**Methods:**

Aeropollen samples were collected for 12 months at the Faculty of Science, Mahidol University in Bangkok, using a RotoRod Sampler^®^. For the investigation of diurnal effect, pollen was collected at 7 a.m., 10 a.m., 1 p.m., 4 p.m., and 7 p.m. For the study of height effect, data were collected at 2, 10, and 18 meters above ground.

**Results and discussion:**

This is the first study of the effects of diurnal cycle and height variation on airborne pollen count in Southeast Asia. The results showed the highest concentration of aeropollen was observed in November, which was at the beginning of the northeast monsoon season in Bangkok, whereas the lowest concentration was recorded in July (rainy season). Interestingly, the lowest airborne pollen concentration recorded in July was greater than the high level of most standards. Grass pollen was found as the major aeropollen. The highest total pollen concentration was detected at 1 p.m. The maximum pollen quantity was detected at 10 meters from the ground. However, the total aeropollen concentration was extremely high (>130 grains/m^3^) at all elevated heights compared to other studies that mostly found at lower height (approximately 1–2 m above ground). The result suggested that pollen concentrations of most pollen types increased as height increased. This study also illustrated the correlation between aeropollen quantity and local meteorological factors.

**Conclusion:**

This aeropollen survey reported that pollen concentration and diversity were affected by seasonal variation, diurnal cycle, and height from the ground. Understanding these relationships can help with predictions of aeropollen type and quantity.

## 1. Introduction

Airborne pollen is recognized as one of the high potential sources of outdoor aeroallergen inducing respiratory allergic diseases, particularly allergic asthma and rhinitis. Pollen allergies affect ~10–30% of the global population (1, 2). Therefore, atmospheric pollen monitoring in view of diurnal and seasonal fluctuations is required as an indicator for allergic patients to be aware of the airborne pollen exposure, especially at the peak of pollen distribution.

Three common sources of airborne allergenic pollen include trees, weeds, and grasses. In temperate and Mediterranean zones, a large percentage of the population are sensitized to weed (e.g., ragweed) and tree (e.g., birch, olive, and cypress) pollen (3–5). Worldwide, grass-family (Poaceae) pollen is the dominant and influential type causing hospitalization due to asthma and allergy, particularly in Australia, Turkey, China, and also Thailand (6–12).

Several studies of pollen diversity illustrated the variation of dominant airborne pollen types collected from different regions and in different seasons. Countries/cities that reported tree pollen as the dominant type during pollen season include Japan (e.g., Japanese cedar and Japanese cypress), Shanghai (e.g., Broussonetia), and most parts of America (e.g., oak, ash, and birch) (13–15). On the other hand, grass pollen is dominant in Portugal, North–West Turkey, West Bengal (India), South Korea, and Thailand (16–20). Furthermore, most of these pollen surveys reported seasonal and annual variations of dominant airborne pollen types. These variations warrant that pollen surveys be performed locally and preferably constantly, to better understand the actual pollen exposure of patients in each specific locality and season.

However, constant pollen monitoring is not possible in certain regions where resources are limited. Under such circumstances, it is imperative that factors influencing pollen count such as seasonality, weather conditions, diurnal cycle, and height from ground, be investigated. Previously, the study of diurnal variation of pollen in London showed the maximum concentration of pollen in late afternoon and early evening (21). Maximum pollen detection could also be dynamic due to the factors of elevated temperature and wind direction (21). Based on a 2-year aerobiological survey in India, the amount of airborne pollen showed a positive correlation with temperature, while negatively correlated with levels of rainfall and relative humidity (19). Likewise, the total airborne pollen concentration in North–West Turkey significantly decreased in both the rainy season and high relative humidity period (20). Airborne pollen collected from the Beijing Museum of Natural History, China, at the height of 10 m above ground level confirmed that the quantity of airborne pollen was affected positively by temperature, but negatively by relative humidity and rainfall (22, 23). On the contrary, a higher amount of airborne pollen was detected on days with high relative humidity, particularly rainy days in summer in the East-Mediterranean coast of Turkey (6). Wind speed is positively correlated with the pollen concentration of the Urticaceae family but not Fagaceae (20). This evidence suggested that the amounts of pollen could be affected by wind and not all plant families or species were affected in the same way (6).

Airborne allergen has been studied mostly in Europe, America, and Mediterranean areas (6, 24, 25). In Southeast Asia, including Thailand, only a few reports of airborne pollen are available, none encompass different factors affecting airborne pollen patterns. Knowledge about seasonal and diurnal fluctuations of airborne pollen in this area is vital because the population of Southeast Asia accounts for more than 8.6% of the world population, which is almost as large as the European population and almost twice the size of the North American population (26). Moreover, urbanization in this region is increasing rapidly, leading to higher prevalence of allergic rhinitis.

The aim of this study was to investigate the dynamics of airborne pollen in terms of pollen types and amounts through seasonal variation, diurnal cycle, and different heights from the ground, in Bangkok, Thailand as a representative of metropolitan areas of Southeast Asia.

## 2. Materials and methods

### 2.1. Sampling area and pollen collection

Airborne pollen samples were collected using the RotoRod Sampler^®^ Model 20 (Multidata LLC, MN, USA) at Mahidol University in Phayathai district [LAT/LONG: 13.765305, 100.526285], within the metropolitan area of Bangkok. The sampler was calibrated using a stroboscopic tachometer following the manufacturer (27). Collector rods were prepared and proceeded under standardized conditions (28). For the seasonal variation study, the sampling device was placed at an open-air walkway between two buildings elevated 10 m above ground, 1 day every week except for the last week of the month for 12 months at 10 a.m. To study the effect of time of day, samples were collected at 7 a.m., 10 a.m., 1 p.m., 4 p.m., and 7 p.m. To investigate the influence of height on pollen concentration and diversity, pollen samples were collected from 10 a.m. to 11 a.m. at 2, 10 and 18 m above ground. Each collection had the duration of 1 h.

### 2.2. Pollen identification and counting

The exposed rods were placed onto a holder slide and stained with Calberla's solution as mounting media. Our rod readers were trained by a set protocol and passed our internal standard testing with <20% difference between readers. Pollen grain types were identified based on their physical characteristics under a compound light microscope (Olympus Biological Microscope Model CX31, Olympus Corp., PA, USA) at 40× magnification. For each collection session, only pollen grains within the area imposed by the 22 × 22 mm cover glass on the surface of one (randomly chosen) of two rods were counted. The amount of pollen was converted to pollen concentration (c) in cubic meters (m^3^) following the RotoRod Sampler^®^ instruction as shown in Equation [1]. In order to calculate the pollen concentration, volume of air sampled by the rods (v) was needed, which was calculated using Equation [2] with the following parameters: rod area (calculated from 0.03 × 0.015 m), diameter of RotoRod Sampler^®^ head (*d*) (0.095 m), revolution per minute (2,400 rpm) and minutes sampled per day (*t*) (60/1,440 min). According to the RotoRod Sampler^®^ operating instruction, the volume of air, which is computed with 60 min sampling time is equal to 0.013 m^3^. Therefore, the final equation used to evaluate pollen concentration is given in Equation [3].


(1) $$\begin{array}{lll} \text{c } & = & \;\frac{\text{total number of pollen grains counted}}{\text{volume of air sampled by the rods}}\; \end{array}$$

(2) $$\begin{array}{lll} \text{v } & = & \;\left(\text{rod area}\right)\text{ x }\left(\text{d x }\pi \text{ x rpm x t}\right)\; \end{array}$$

(3) $$\begin{array}{lll} \text{c } & = & \;\frac{\text{total number of pollen grains counted}}{0.013\;{\text{m}}^{3}} \end{array}$$

### 2.3. Meteorological data

Bangkok climate has been classified as a tropical savanna climate under the Köppen climate classification. Three seasons can be found: rainy or southwest monsoon season (mid-May to mid-October), winter or northeast monsoon season (mid-October to mid-February) and summer or pre-monsoon season (mid-February to mid-May) (29). In general, surface temperature is high with the hottest period of the year in March to May, when maximum temperatures near 40°C. The northeast monsoon season is mild, with an average temperature of 26°C. The prevailing winds in Bangkok during the northeast monsoon season are mostly north and northeast. The average rainfall is approximately 1,200 mm per year. In this study, meteorological data, including daily relative humidity, wind speed, rainfall and temperature, were collected at Klong Toey (Station Code 455201) [LAT/LONG: 13.704610, 100.575534] situated ~4 km from the pollen collection site were obtained from the Thai Meteorological Department.

### 2.4. Statistical analysis and data illustration

Pollen concentration and meteorological data were analyzed and illustrated using GraphPad Prism version 9.0.0 (GraphPad Software Inc., CA, USA). The assumptions of normality and homogeneity of variance were tested using Shapiro-Wilk test and Levene's test, respectively. Spearman's rank correlation was applied in order to assess the correlation coefficient between dynamics of pollen concentration and meteorological factors over the study period. Statistical significance was determined at the level of 0.05.

## 3. Results

Airborne pollen in Bangkok was identified based on morphological characteristics as members of six major plant families: Poaceae, Arecaceae, Typhaceae, Casuarinaceae, Caesalpinioideae, and Amaranthaceae. Pollen from less dominant plant families were grouped together and referred to as “Others,” namely Acanthaceae, Cyperaceae, Mimosoideae, Asteraceae, Cycadaceae, Pinaceae.

The most abundant pollen type collected was Poaceae or grasss pollen (32.86%) with an average of 53 grains/m^3^ with the maximum count in November (446 grains/m^3^) (Table 1). Poaceae pollen was also ranked first in terms of frequency of occurrence (0.83), calculated from the number of rods contained each pollen type divided by total number of sampling rods. Even though Typhaceae pollen was ranked third in average concentration (25 grains/m^3^), its frequency of occurrence was relatively high (0.63), second only to Poaceae pollen. A number of pollen grains could not be identified due to high plant diversity in the area and lack of an extensive pollen reference database. Together the unidentified pollen averaged at 100 grains/m^3^ and its highest concentration appeared in February (1,815 grains/m^3^). Because of the non-uniform nature of the unidentified pollen, it was excluded from further analyses.

**Table 1:** Variation of airborne pollen concentration throughout the year of this study^†^.

**No**.	**Pollen type**	**Avg conc. (pollen grain/m^3^)**	**Max conc. (pollen grain/m^3^)**	**Month of max conc**.	**Percent of total^††^**	**Frequency of occurrence^†††^**
1	Poaceae	53	446	Nov	32.86	0.83
2	Arecaceae	28	1,215	May	17.65	0.35
3	Typhaceae	25	523	Sep	15.50	0.63
4	Others	22	184	Nov	14.03	0.18
5	Casuarinaceae	16	115	Mar	9.99	0.58
6	Caesalpinioideae	8	184	Mar	5.32	0.36
7	Amaranthaceae	7	176	Sep	4.64	0.32

† Pollen samples collected 1 day every week except the last week of the month for 12 months at 10 m above ground and collected at 10 a.m.;

†† calculated from the concentration of each pollen type divided by total pollen concentration and converted to percentage;

††† calculated from the number of rods contained each pollen type divided by total number of sampling rods.

**Figure 1:**
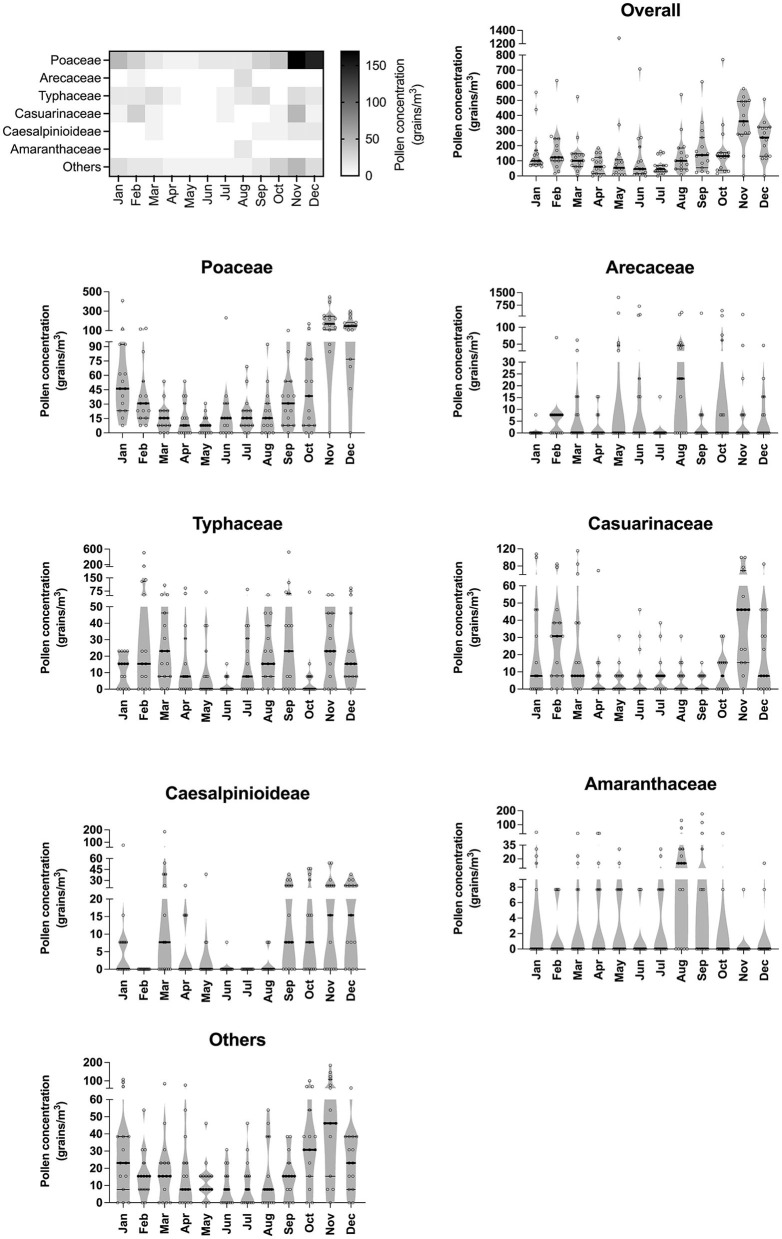
Variations of pollen quantity throughout seasons. Heat map generated based on the median of each pollen species illustrates airborne pollen distribution throughout the year. Error bars of violin plots represent median and interquartile range.

Seasonal variation of airborne pollen was investigated by analyzing pollen types collected 1 day each week for every week of the month except the last month throughout 12 months at 10 a.m. Pollen dynamics were analyzed together and independently for each pollen type. Average pollen concentration and maximum values varied by months (Figure 1). Overall, the total pollen concentration began to increase in August (pre-monsoon season), reaching the maximum concentration in November (early northeast monsoon) before decreasing in January (late northeast monsoon). The first peak of average pollen concentration was recorded in November at the average concentration of 360 grains/m^3^ (median: 362 grains/m^3^) and the second peak was found in December at 241 grains/m^3^ (median: 254 grains/m^3^). Low pollen concentration was detected in April (mid-summer) at an average of 74 grains/m^3^ (median: 62 grains/m^3^) and July at an average of 62 grains/m^3^ (median: 465 grains/m^3^). Poaceae pollen was abundantly distributed in the atmospheric air over the entire period of collection. The variability of monthly pollen concentrations is demonstrated in Figure 1. The peak average pollen counts of Typhaceae and Caesalpinioideae were recorded in the first trimester (January–March) while the maximum average concentrations of Poaceae, Others, Casuarinaceae, and Amaranthaceae were observed in the third-to-last trimester (September–December).

**Figure 2:**
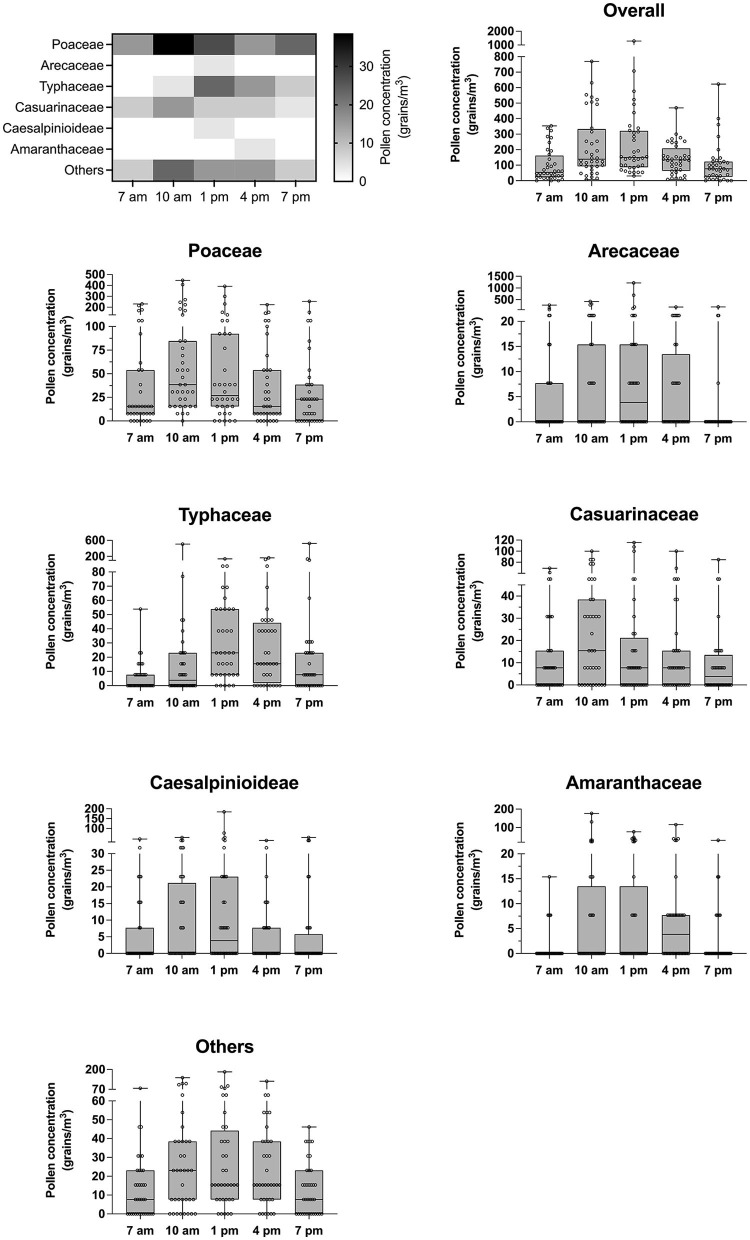
Deviations of pollen count influenced by diurnal cycle. Heat map generated based on the median of each pollen species illustrates airborne pollen fluctuation at each diurnal cycle. Error bars of box plots represent median and interquartile range.

Considering the diurnal distribution patterns of the pollen types studied, certain differences were observed. Overall, the highest peak of average pollen concentration was found in the early afternoon (1 p.m.) whereas the lowest concentration was recorded in the early morning (7 a.m.) (Figure 2). Two patterns of hourly distribution could be observed. First, consisting of Poaceae, Casuarinaceae, Amaranthaceae, and Others, maximum average pollen was found at 10 a.m. and started to decline until the lowest count was reached at 7 p.m. In the second group, Arecaceae, Typhaceae and Caesalpinioideae pollen types started to increase in the late morning hours reaching the maximum value in the early afternoon (1 p.m.) before gradually decreasing at 4 p.m. until 7 p.m. The maximum average concentration of Arecaceae pollen (mean: 69 grains/m^3^; median: 4 grains/m^3^) was approximately 2- and 4-fold higher than the concentration of Typhaceae (mean: 33 grains/m^3^; median: 23 grains/m^3^) and Caesalpinioideae (mean: 17 grains/m^3^; median: 4 grains/m^3^) pollen types, respectively.

**Figure 3:**
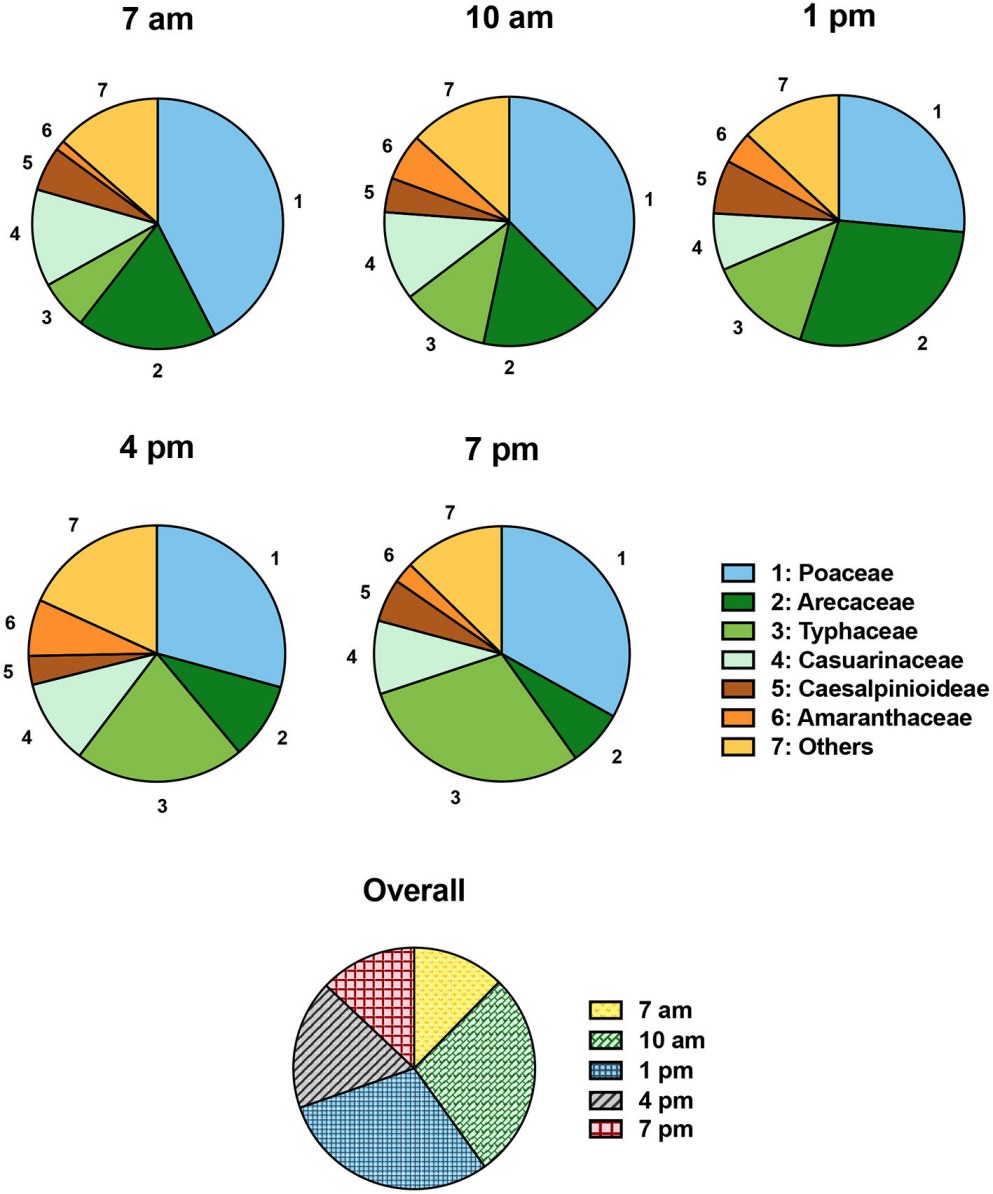
Compositions of individual pollen type during each diurnal cycle.

The overall pollen composition was similar among time points during each diurnal cycle (Figure 3). In the morning, at 7 and 10 a.m., Poaceae pollen dominated approximately one half of the total pollen quantity. At 1 p.m., two pollen types: Poaceae and Arecaceae together contributed slightly over half of all pollen counts. In the late afternoon to early evening (4–7 p.m.), the dominant pollen type was Poaeceae, accounting for about one third of total pollen count.

**Figure 4:**
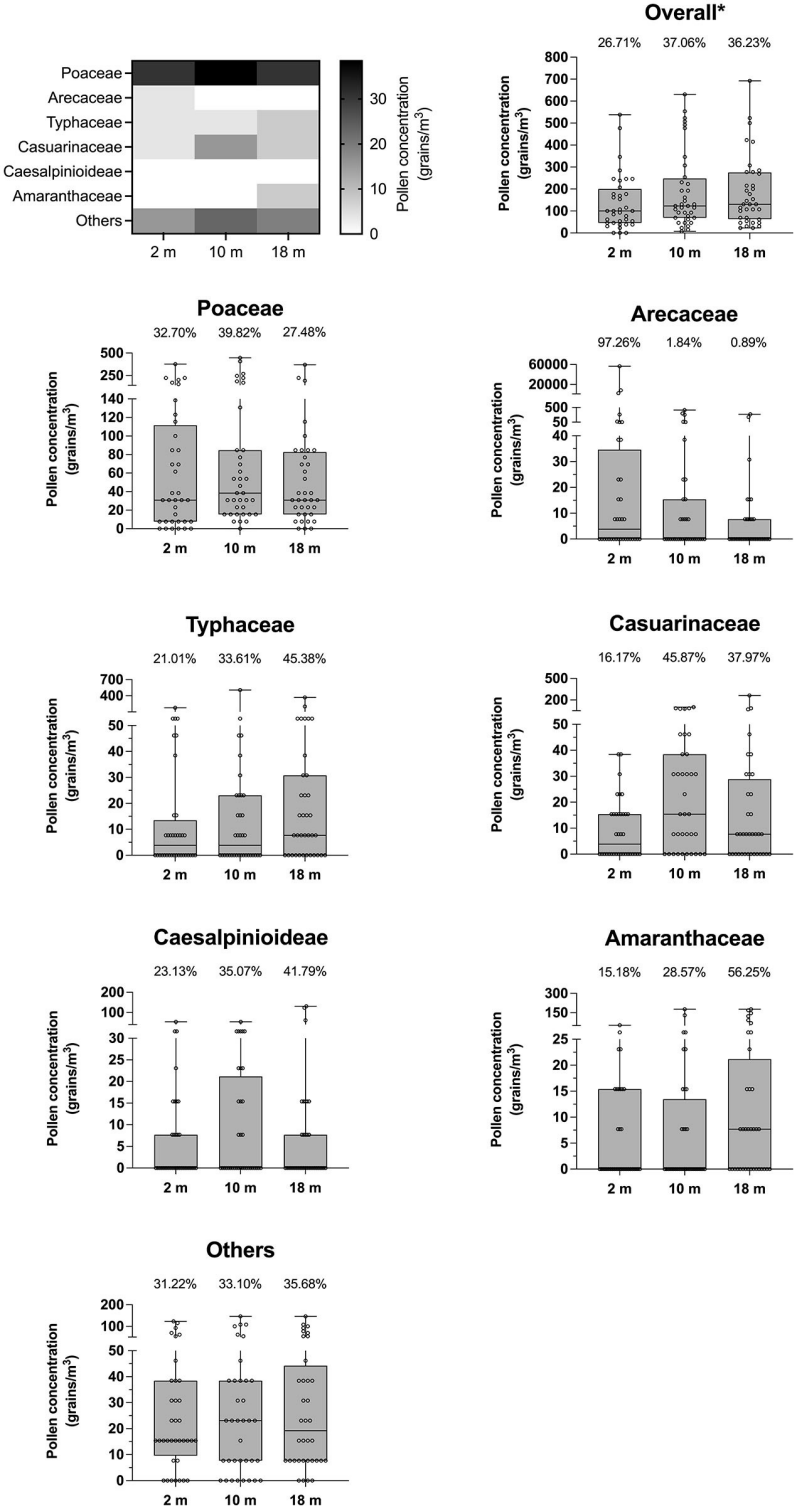
Dynamics of pollen concentration in relation to height from ground. Heat map generated based on the median of each pollen species illustrates airborne pollen dynamics at different heights. Error bars of box plots represent median and interquartile range. A number above each bar represents the percentage of pollen concentration at each height divided by total pollen concentration of each pollen type. *Overall pollen counts excluded the amount of Arecaceae pollen.

Variations in all pollen types at the three levels of height (2, 10, and 18 m above ground) were observed (Figure 4). The total airborne pollen concentration was maximal at 10 m above ground with an average of 191 grains/m^3^ (median: 123 grains/m^3^). This reported concentration was defined by excluding the Arecaceae (palm) pollen count (mean: 1,884 grains/m^3^; median: 4 grains/m^3^) due to its extremely high concentration in a short period with non-normal distribution. Minimal concentrations were found at 2 m (mean: 137 grains/m^3^; median: 100 grains/m^3^), and at 18 m (mean: 186 grains/m^3^; median: 131 grains/m^3^). The pollen of Poaceae was dominant at all three heights. Typhaceae, Casuarinaceae, Caesalpinioideae, Amaranthaceae, and Others pollen types presented a moderate-to-low average concentration over the three levels. Only Arecaceae showed the exceeding average pollen concentration recorded at 2 m and sharply declined at 10 and 18 m. Poaceae, Casuarinaceae and Caesalpinioideae pollen was most concentrated at 10 m, whereas the peak pollen counts of Typhaceae and Amaranthaceae were recorded at 18 m. Of note, the pollen concentration of Amaranthaceae doubled at each height.

**Figure 5:**
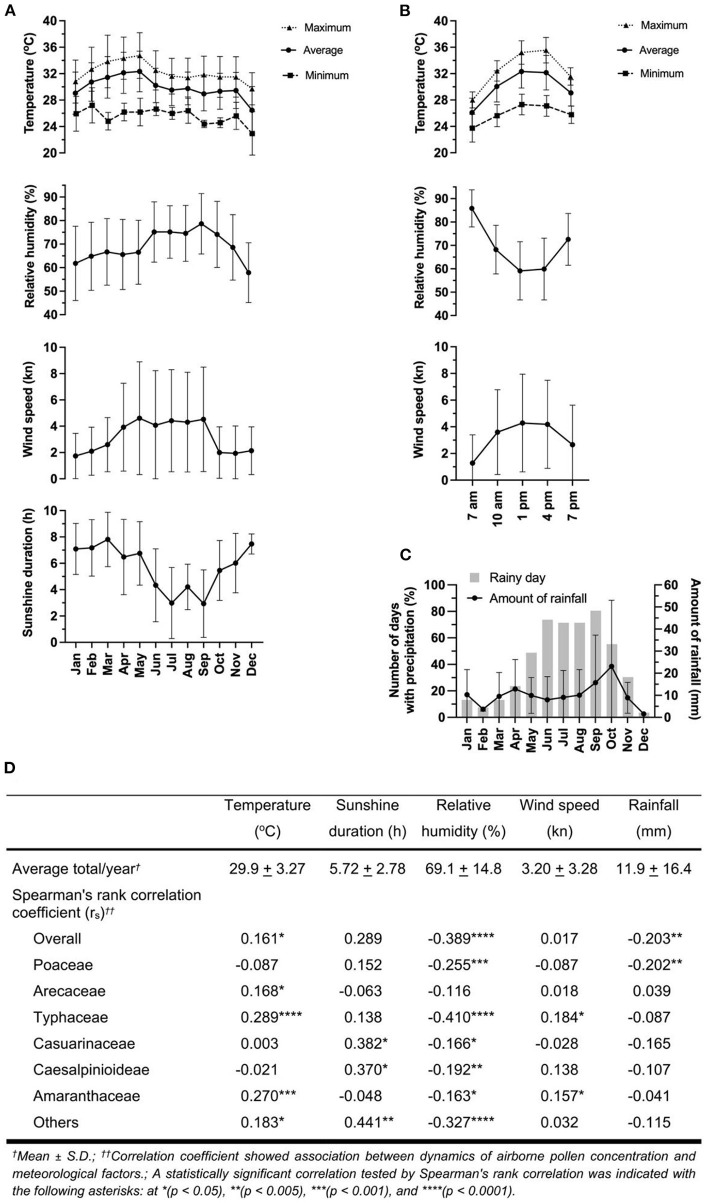
Meteorological data, total pollen concentration and their correlation. Meteorological records by **(A)** month and **(B)** time of collections including temperature, relative humidity, wind speed, and sunshine duration. **(C)** Amount of rainfall and number of days with precipitation. **(D)** Correlation analysis of meteorological variations and atmospheric pollen concentrations using Spearman's rank correlation at a significant level of 0.05. Error bars represent standard deviation (SD).

Meteorological records during the study period were analyzed to gain a better understanding about their relationship with pollen count. Meteorological parameters consisted of monthly and intradiurnal average of temperature (degree Celsius, °C), relative humidity (%), wind speed (knot, kn), and the average duration of sunshine (hour, h) (Figures 5A,B). The amount of rainfall (milliliter, mm) (Figure 5C) was obtained from the recorded data each month. Spearman's rank correlation (non-parametric correlation) analysis was performed to test the association of the pollen concentration and the meteorological data. The overall pollen concentration showed a negative correlation with relative humidity (*r*_*s*_ = −0.389, *p*-value < 0.0001) and amount of rainfall (*r*_*s*_ = −0.203, *p*-value < 0.005), whereas a positive correlation was found with temperature (*r*_*s*_ = 0.161, *p*-value < 0.05) (Figure 5D). The negative correlation between pollen concentration and relative humidity was statistically significant for all pollen types except Arecaceae. Pollen concentration was also found to be negatively correlated with rainfall for Poaceae. Temperature had a significant positive correlation with pollen counts of Arecaceae, Typhaceae, Amaranthaceae, and Others. Sunshine duration was found to be strongly correlated with pollen counts of Casuarinaceae, Caesalpinioideae, and Others; while wind speed had a positive correlation with Typhaceae and Amaranthaceae pollen counts.

## 4. Discussion

As of 2019, an up-to-date study of allergic rhinitis (AR) reported slightly higher incidences (13–24%) in Thai children than the average Asia-Pacific prevalence (6–15%), and global prevalence (9–16%) (30). According to several previous studies, bioaerosols, especially airborne pollen, have confirmed the remarkable clinical impact to cause severe allergic respiratory diseases (31). Allergies such as pollen allergies are increasing also due to urbanization and climate change (32). Therefore, updated information about atmospheric pollen, particularly types and concentrations throughout diurnal cycles and seasons in each locality is much-needed. Due to a number of limitations, aeropollen surveys from countries in tropical/subtropical regions, including Thailand, are not frequently reported.

This study provides the information about airborne pollen dynamics, especially quantity and diversity affected by diurnal and seasonal fluctuations as well as different heights above ground. The experimental site was chosen because it has an open walkway at different heights appropriate for the study design and situated in an area not significantly different from other Bangkok metropolitan areas in terms of vegetation or meteorological factors. The highest concentration of total pollen at 446 grains/m^3^ was found in November, consistent with previous studies from this region with the maximum pollen concentration during September to February (18, 33). The lowest total concentration of airborne pollen was observed in July at an average <65 grains/m^3^. The pollen counts in all other months were >70 pollen grains/m^3^. Interestingly, the lowest airborne pollen concentration recorded in July was even greater than the high level of most standards (34). The standard of recommendation to categorize pollen levels reported from the Scottish Center for Pollen Studies, for example, was divided into 3 groups including low (≤50 grains/m^3^), medium (51–100 grains/m^3^), and high (>100 grains/m^3^) (34). Compared to previous surveys in Bangkok in 1980 and 2012–2013, the recorded monthly concentrations in this study were similar (18). This observation suggested that people who lived in Bangkok have been exposed to an extremely high level of aeropollen year-round.

Airborne pollen recorded in this survey was identified as members of six heterogenous plant families. Aeropollen from less dominant plant families were grouped together and refered to Others. Among the collected pollen, Poaceae was the most dominant throughout the year. Typhaceae and Amaranthaceae were also previously found to rank among the top-5 airborne pollens in Bangkok. Interestingly, these three allergenic pollen were predominant in the northeast monsoon season (September–February), congruent with that reported in the previous study (18). These results strongly suggest that atopic patients with AR symptoms worsening during these months should be tested for pollen sensitization, especially Poaceae, Typhaceae, and Amaranthaceae pollen.

Interestingly, the total pollen counts from 7 a.m. to 7 p.m. showed almost equal distribution in all time points, although the highest average concentration was found at 1 p.m. According to previous studies, maximum pollen count was found in late morning between 9 and 11 a.m., together with gradual increase of temperature, and gradual decrease of relative humidity and wind speed (19, 35). Several studies in the Americas and Europe showed that maximum concentrations of most pollen types were recorded at midday or noon (21, 36, 37). This difference is speculated to be due to the effects of local flora combined with temperature and wind direction to drive the pollen distribution from pollinating trees to the collection points (21). Although the diurnal cycles of anther opening are genetically determined, the hourly dynamics of atmospheric pollen load, in particular, is related to meteorological parameters in each season of individual study sites (38, 39). In addition, the number of airborne pollen grains is also related to pollination timing. The alder (*Alnus*) pollen counts in two different climate regions of Poland, namely Rzeszów and Szczecin, showed that the highest quantity of alder pollen in Rzeszów was found at the beginning of pollination while high pollen count in Szczecin was recorded throughout the pollination period. Significantly, the timing of pollination is also related to meteorological factors. For example, the pollination timing could be delayed as a result of low temperature and solar radiation, but high velocity wind can lead to changes in diurnal rhythms of airborne pollen concentration (40).

Temperature, relative air humidity, precipitation, wind speed, and total sunshine duration are recognized as the important meteorological parameters that determine aeropollen concentration (41, 42). Temperature is the most important factor influencing variations in airborne pollen concentration. It seems evident that higher temperatures accelerate the process of ripening of the flowers, and, consequently, the starting dates of the pollen season will be earlier. However, the effect of temperature on these processes is complex, and other environmental factors may be of great influence (43). Consequences of global climate change are structured into two main categories: direct effects (an extreme increase of average temperature) and indirect effects (such as disturbances of ecological systems and biological effects including alteration of pollen release) (44). A study from Iowa, USA demonstrated that the delayed occurrence of daily airborne pollen shed peak in 2004 (after 10 a.m.) compared to 2003 (at 9–10 a.m.) was a result of lower average air temperature in 2004 (45). Likewise, a study from the Netherlands reported a strong correlation between temperature and timing of the start of pollen season. The study showed the start of pollen season began almost 20 days earlier in the 1990s compared to the 1970s, and a simulation estimated that the pollen season will start up to 23 days earlier in the 2090s compared to the 2000s. This result suggested that the earlier start of pollen season will have consequences for hay fever season (46). Apart from the effect of temperature on pollen season shift, high air temperature also affects daily pollen concentration in each area. For example, a positive statistically significant correlation between average temperature and daily alder pollen count was shown in the study from Poland (47). A 20-year study of meteorological variables and pollen dynamics in Raleigh, North Carolina, a humid subtropical climate, revealed a strong positive correlation between temperature and airborne pollen concentration, particularly tree pollen (48). Our study also showed that temperature positively correlated with pollen count, especially with the weed pollen types (Typhaceae and Amaranthaceae).

Temperature and carbon dioxide (CO_2_) concentration are rising globally. In Japan, the temperature has increased by an average of 1.16°C in the past century (49, 50). As a result of the elevation of temperature and CO_2_ concentration, metabolic processes including water uptake and photosynthesis in pollen-producing plants are increased, consequently, promoting pollen production (51). Moreover, airborne pollen concentration positively correlates with sunshine duration as shown in several previous studies. Daily alder pollen counts in Poland and cedar and cypress pollen counts in Japan were also shown to have a positive association with sunshine hours (47, 50). Similar to our study results, sunlight hour was positively correlated with the Casuarinaceae and Caesalpinioideae pollen types, which mainly consist of shrubs and trees. For these two groups, there was a minor peak of pollen concentration in February to March, which was likely contributed by species with long-day flowering habits. In this survey, the maximum concentration of total aeropollen was recorded in November, in which short-day plants respond to an increase in temperature and decreased daylength by flowering and releasing pollen grains. The highest count of total airborne pollen could also be due to other meteorological parameters such as low wind speed and relative humidity as well as less rainfall. Long sunshine duration can also impact pollen dispersion. For temperate regions, higher temperature and sunnier climates can be exposed to ultraviolet (UV) radiation for longer periods, resulting in higher pollen counts because UV light is likely to influence vegetative growth and flower initiation (52, 53). However, in the subtropical/tropical regions, extremely high ambient temperatures could also inhibit flowering, as seen in April in our study.

Previous studies showed that airborne pollen loads were low when the amount of precipitation was high (47, 54). In concordance with the previous studies, our study found that pollen concentration was negatively correlated with relative humidity and rainfall. After the southwest monsoon season (June–October), the relative humidity dropped and the overall airborne pollen concentration obviously increased. The overall pollen concentration peaked in the month of decreased days with precipitation and amount of rainfall (November). This phenomenon could be due to several reasons. First, high relative air humidity during precipitations restricts anther opening, leading to low amounts of pollen release. Second, highly hygrophilous cells of pollen grains are rapidly hydrated under high humidity and consequently increasing their weight and depositing them on the ground by gravity (20). High relative air humidity during precipitations restricts the anther opening leading to low amounts of pollen release (54). In addition, rainfall collects pollen grains suspended in the air leading to their agglomeration, followed by falling to the ground resulting in the decrease in atmospheric airborne pollen concentration (55). Third, airborne pollen could completely rupture and release cytoplasmic contents upon hydration, and thereby could not be counted in pollen surveys. The pollen rupture could be highly synchronized and releases allergenic content simultaneously, leading to the thunderstorm allergy and asthma attacks (56, 57). The pollen grains and allergenic particles on the ground can become airborne again *via* a redeposition process by the wind (54). Apart from the effects of wind and precipitation on pollen load, the extreme or unusual weather conditions such as strong wind or heavy rain was a limitation in this study. In such events, pollen collection was shifted to the following day to reduce the outlier data points.

Height from the ground could affect variation of aeropollen. Accurate counts of local pollen can be obtained at a lower height, whereas the study of daily pollen dispersion is more appropriate at a greater height (58). Generally, most airborne particulates are measured at approximately 10–30 m above ground level (59). However, several previous studies reported that pollen quantity and/or diversity at nearly ground level was higher than at elevated heights. For example, a study from mainland China revealed that the highest concentration of both total annual and average daily pollen was significantly found at 1.5 m above ground compared to 35–70 m (60). Routine monitoring of aeroallergens in Finland found that daily concentrations of most pollen types such as grass and weed was higher at ground level than at roof level—about 15 m above ground (59). Likewise, a study from Spain showed greater heterogeneity of airborne pollen types at 1.5 m (average human height) compared to 15 m (standard sampling height) (58). In this study, we found the highest pollen concentration of Arecaceae at 2 m above ground, where most human exposure occurs, and the quantity of these pollen types declined gradually with increasing height. Conversely, the counts of other dominant pollen types including Typhaceae, Amaranthaceae, and Caesalpinioideae increased with increasing height and we found the peak concentration of these three pollen types at 18 m from the ground. This result could be predominantly related to the quantity of big- and medium-sized airborne pollen, which are big particulates with a large mass and a high settling velocity, and, as consequence, the pollen concentration at lower height is greater (60, 61).

Apart from the effect of pollen size, the prevalence of airborne pollen types is related to local vegetation sources (23, 62). The high concentration of Arecaceae (palm) pollen in this study was not reported in previous airborne pollen surveys in Bangkok, which was most likely a result of the existence of Arecaceae trees nearby the sampling area. The Arecaceae pollen concentration declined sharply as height increased, suggesting that its dispersion was likely limited to local areas. In addition, the grain size of Aracaceae pollen is larger, further supporting that this pollen type cannot travel long distances. The concentration of predominant pollen type such as Poaceae (grass family) was recorded at 10 m above ground, possibly because physical barriers at the ground/near-ground level including trees and buildings interrupted the dispersion of these pollen types into the elevated height. In reality, the local newly dispersed pollen, pollen from a distant location, and re-distributed pollen all contributed to the total pollen concentration at a given time. The fact that we observed pollen peak time for almost all species suggests that diurnal cycle was a major factor of the pollen concentration, and that local vegetation is likely the key contributor. Importantly, this study and previous studies suggest that living at greater heights does not necessarily reduce pollen exposure. Therefore, patients sensitized to pollen who live in high-rise buildings should be recommended to take the same precautions as those living near ground level.

## 5. Conclusion

In conclusion, this study reported an aeropollen survey in Bangkok, the capital city of Thailand. The peak pollen concentrations were recorded in November and December, while the lowest pollen count was found in July. The dominant pollen types were Poaceae followed by Arecaceae and Typhaceae. The average pollen quantity was highest from late morning (10 a.m.) to early noon (1 p.m.). The highest pollen concentration was observed at 10 m above ground. This study also illustrated the correlation between airborne pollen quantity and local meteorological factors. Understanding these relationships can help with predictions of airborne pollen type and quantity.

## Data availability statement

The original contributions presented in the study are included in the article/supplementary material, further inquiries can be directed to the corresponding author.

## Author contributions

WS: conceptualization, supervision, and funding acquisition. WS, YJ, SS, and US: methodology, investigation, and writing—review and editing. YJ and SS: data curation. WS and YJ: writing—original draft preparation and visualization. All authors contributed to the article and approved the submitted version.
